# Vigabatrin-Associated Brain Abnormalities on MRI in a Patient with PCDH19-Clustering Epilepsy

**DOI:** 10.3390/jcm15124619

**Published:** 2026-06-14

**Authors:** Olena Apanasenko, Ewelina Głodek-Brzozowska, Paweł Guz, Agnieszka Łobodzińska, Lidia Perenc

**Affiliations:** 1 Clinic of Pediatric Neurology and Pediatrics, St. Jadwiga the Queen Voivodeship Clinical Hospital in Rzeszow, Lwowska 60, 35-301 Rzeszow, Poland; ewe.brzozow19@gmail.com (E.G.-B.); 2 Faculty of Medicine, Collegium Medicum, University of Rzeszow, ul. Warzywna 1a, 35-310 Rzeszow, Poland; 3 Clinical Department of Radiology and Imaging Diagnostics, St. Jadwiga the Queen Voivodeship Clinical Hospital in Rzeszow, Lwowska 60, 35-301 Rzeszow, Poland; pguz@op.pl; 4 MedGen Medical Centre, Wiktorii Wiedenskiej 9a, 02-954 Warsaw, Poland; agnieszka.lobodzinska@medgen.pl

**Keywords:** PCDH19-clustering epilepsy, infantile spasms, whole-exome sequencing, vigabatrin, VABAMR, carnitine deficiency, case report

## Abstract

**Background**: Cluster epilepsy related to PCDH19 is a rare X-linked disorder that mainly affects females. Atypical presentations, such as infantile spasms, are exceptionally rare, leading to diagnostic and therapeutic challenges. **Case Summary**: An 8-month-old girl presented with early-onset, drug-resistant epilepsy from 4 months of age, displaying tonic and focal seizures, infantile spasms with hypsarrhythmia, and neurodevelopmental regression. Whole exome sequencing identified a novel heterozygous mutation, c.1072del (p.Val358SerfsTer10), in the PCDH19 gene. Following vigabatrin therapy for infantile spasms, the patient subacutely developed a movement disorders: dystonic movements and action tremor in forearm and hands. Brain Magnetic Resonance Imaging (MRI) revealed symmetrical restricted diffusion and cytotoxic edema in both the thalami and the internal capsules, confirming Vigabatrin-Associated Brain Abnormalities on MRI (VABAMR). Concurrently, a systemic carnitine deficiency was identified, which could additionally have compromised mitochondrial bioenergetics and intensified the tendency to cytotoxic cerebral edema. A strategic reduction in vigabatrin led to complete resolution of movement disorder and neuroimaging abnormalities. **Conclusions**: This case underscores the high phenotypic variability of PCDH19 mutations, and also the importance of early advanced genetic testing and the consideration of even rare side effects of drugs in differential diagnosis. It is unclear whether that baseline carnitine deficiency could potentially increase the risk of VABAMR.

## 1. Introduction

Protocadherin 19 (PCDH19)-clustering epilepsy is an X-linked disease, predominantly seen in females [1]. It is one of the most common single-gene disorders responsible for the development of epilepsy in early childhood, with an estimated incidence 1 per 20,600 live born females [2]. The core features of the disease are clusters of seizures, typically induced by fever, that usually start during the first 3 years of life. Intellectual disability and psychiatric symptoms are reported in about two-thirds of patients [1,3].

Structurally, the disease results from a cellular interference mechanism caused by tissue mosaicism in females, which impairs the development of the neural network and down-regulates neurosteroid-mediated GABAergic inhibition (GABA, gamma-aminobutyric acid) [3,4]. The initial electroclinical spectrum in early infancy is usually limited to focal or generalized convulsive seizures, while milestone development prior to onset remains largely unaffected. Atypical initial presentations of PCDH19 mutations, such as a presentation with infantile spasms (IS) or an early evolution into a full-blown Epileptic Spasms Syndrome (IESS), remain exceptionally rare and poorly documented [5]. Such unusual phenotypes pose an immense diagnostic dilemma for clinicians, often leading to significant delays in targeted genetic screening. Furthermore, the standard of care for IESS is heavily based on prompt initiation of vigabatrin therapy, a potent antiepileptic agent. Paradoxically, recent pharmacovigilance and neuroimaging data indicate that vigabatrin can induce transient, drug-related neuroimaging abnormalities (VABAMR, Vigabatrin-Associated Brain Abnormalities in Magnetic Resonance Imaging) and subacute movement disorders in this specific, vulnerable age group [6,7].

The following case report represents diagnostic and treatment difficulties which one may encounter on the way to diagnosis in a patient with early childhood onset epilepsy, underlining the importance of proper genetic testing in this age group, and highlights the critical importance of recognizing treatment-induced iatrogenic complications to prevent misdiagnosis.

## 2. Case Presentation

The 8-month-old girl who was treated for epilepsy with vigabatrin (125–150 mg/kg) and valproic acid (32 mg/kg) was admitted to the neurological department with signs of respiratory tract infection, extreme sleepiness, development regression, movement disorder, and eyelid myoclonia.

The child was born from the second pregnancy complicated by arterial hypertension in the mother, the second natural pharmacologically induced delivery at the 38th week of gestation with birth weight of 3140 g, head circumference of 33 cm and 8/9/10/10 points Apgar score at 1/3/5/10 min. Her newborn screen for inborn errors of metabolism was normal (in Poland a dry blood drop is analyzed using tandem mass spectrometry) [8]. Family’s social history does not indicate the presence of epilepsy, criminal offences, substance abuse, etc. According to the information available, this is the first case of this type in the family.

**Figure 1:**
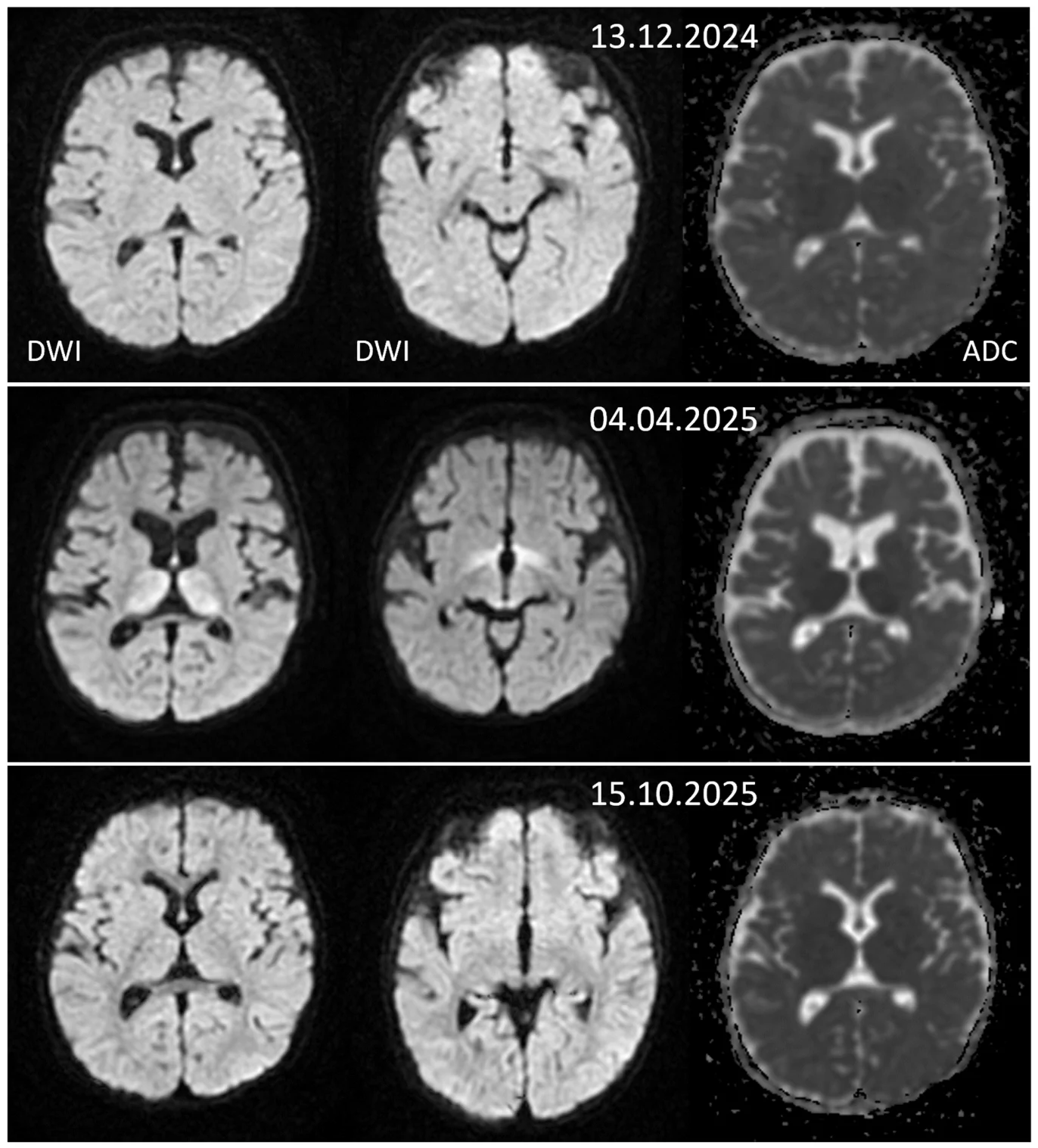
Brain MRI figures performed: at the age of 4 months (13 December 2024), at the age of 8 months (4 April 2025) and at the age of 14 mouths (15 October 2025). Comparison of MRI of DWI/ADC sequences—images from 4 April 2025 showed restricted water diffusion in both the thalami and the lower part of both internal capsules associated with cytotoxic edema in these areas. In the examination from 15 October 2025, these changes resolved. The examination was performed with a GEMS Artist 1.5T device. Abbreviations: MRI—Magnetic Resonance Imaging, DWI—Diffusion-Weighted Imaging, ADC—Apparent Diffusion Coefficient.

At the age of 4 months, multiple paroxysmal events with general stiffening, extension of the head and arms, and clenching of the hands appeared in the child (generalized tonic seizures). At that point, development was normal. The girl smiled socially, gurgled, grasped toys, supported herself with hands in the prone position and turned from the supinated to the prone position with help. The former work-up was performed. Routine laboratory tests were normal. The levels of lactate, ammonia, and urea in serum were also within the reference range. The profile of organic amino-acids in urine was also normal. Interictal Sleep Electroencephalography (EEG) was performed. The graphoelements of sleep were preserved, and few interictal epileptiform discharges were registered on the temporal and parietal leads. Magnetic Resonance Imaging (MRI) of the brain with contrast enhancement revealed only subtle dilatation of the ventricular system with Evance ratio—0.29 (Figure 1). On abdominal ultrasound an anomaly of the left kidney (two pelvicalyceal systems and hydronephrosis) was found. A Next-Generation Sequencing (NGS) epilepsy gene panel was ordered. Meanwhile, the aforementioned paroxysmal events ceased without treatment within 3 days.

At the age of 5 months, the patient returned to the clinic due to frequent focal-impaired consciousness seizures with observable features, and focal to bilateral tonic–clonic seizures. During these events the girl opened her eyes, turned head to the side, then extended and lifted her right arm, next left, occasionally also one of the legs. Sometimes, slight clonic movements were observed at the end of the events. The episodes lasted up to 2 min and were repeated multiple times a day during wakefulness and in sleep.

**Figure 2:**
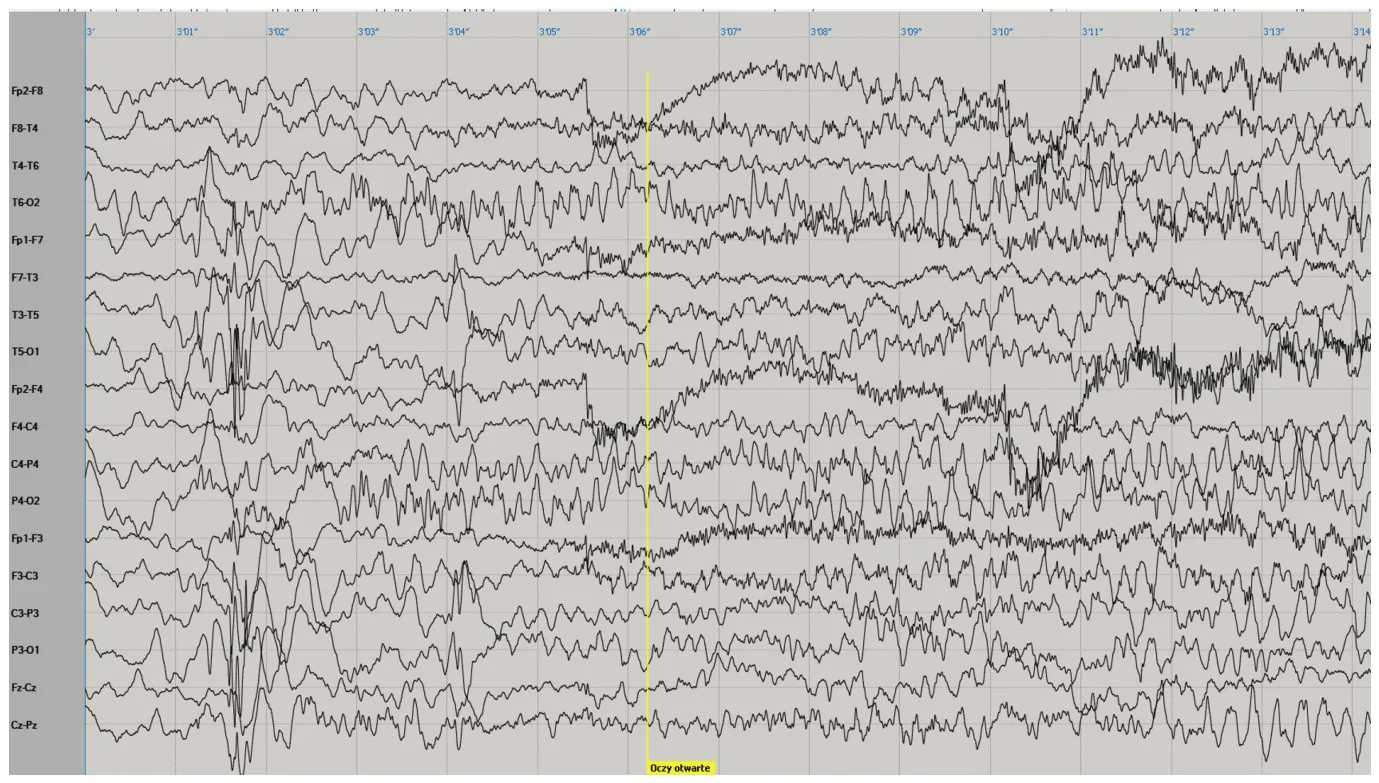
Ictal EEG: 10–20 montage system, bypolar longitudinal montage, sensitivity 10 mcV/mm, LFF 0.5 Hz, HFF 70 Hz, notch filter 60 Hz, paper speed 30 mm/s. Spike-and-wave complexes and polyspikes followed by rhythmic slow wave activity with amplitude up to 320 uV that started over the left temporal region were registered. Abbreviations: EEG—electroencephalography, LFF—low frequency filter, HFF—high frequency filter.

**Figure 3:**
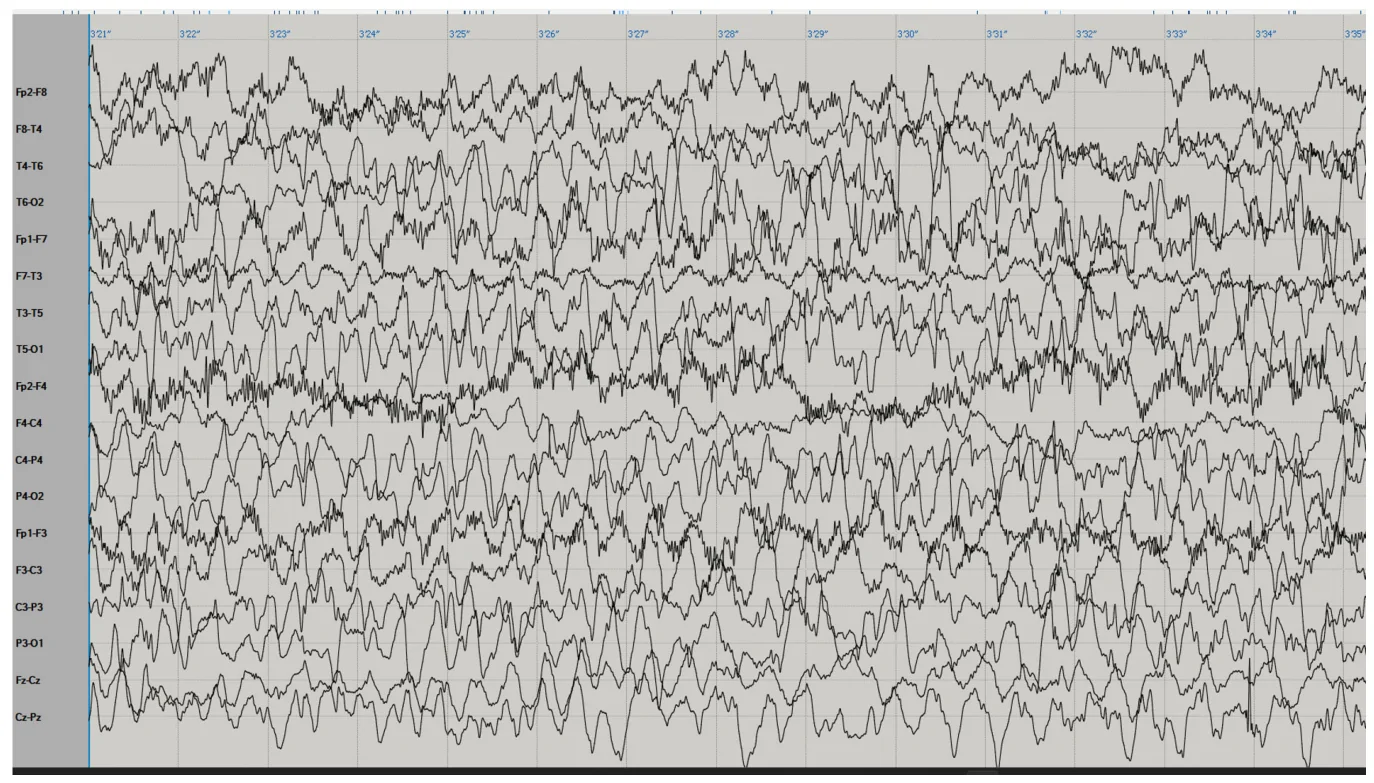
Ictal EEG: 10–20 montage system, bypolar longitudinal montage, sensitivity to 10 mcV/mm, LFF 0.5 Hz, HFF 70 Hz, notch filter 60 Hz, paper speed 30 mm/s. Spike-and-wave complexes and polyspikes followed by rhythmic slow wave activity with amplitude up to 320 uV that started over the left temporal region and propagated over both hemispheres were registered. Abbreviations: EEG—electroencephalography, LFF—low frequency filter, HFF—high frequency filter.

The interictal sleep EEG registered multiple bilateral synchronic spikes and sharp wave—slow wave complexes with amplitude up to 420 μV over the temporo-parieto-occipital regions more prominent on the left side. Ictal EEG registered spike-and-wave complexes and polyspikes followed by rhythmic 3–4 Hz slow wave activity with amplitude up to 320 μV that started over the left temporal region and propagated over both hemispheres during the seizure (Figure 2 and Figure 3). Initiating carbamazepine treatment was ineffective. The patient developed status epilepticus which was ceased by intravenous administration of valproic acid, steroids, and mannitol. Within a few days, the frequency of seizures decreased and finally disappeared. The cluster of seizures lasted 6 days in total.

**Figure 4:**
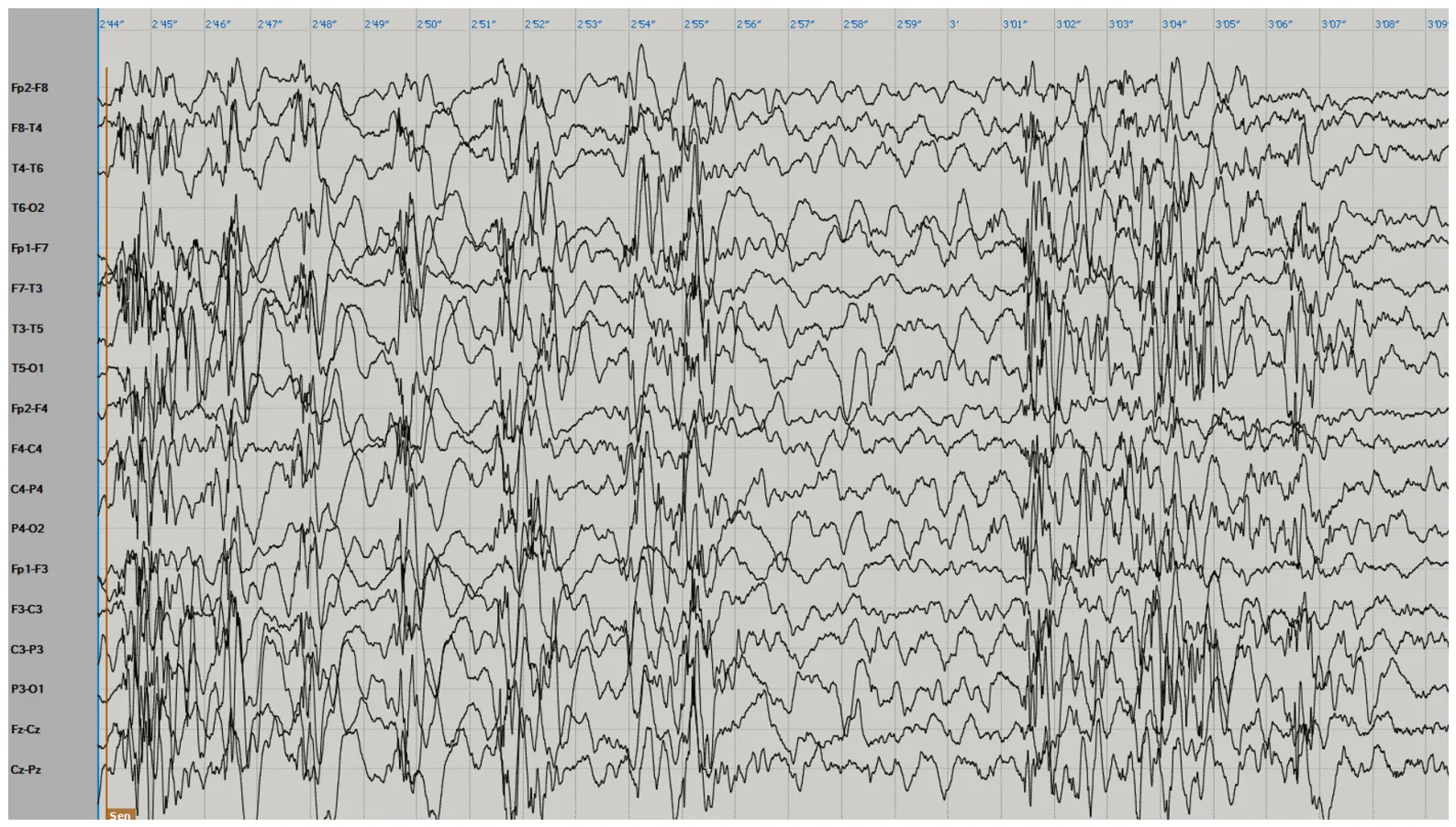
EEG performed in drowsiness at the age of 6 months and 2 weeks: 10–20 montage system, bipolar longitudinal montage, sensitivity 10 mcV/mm, LFF 0.5 Hz, HFF 70 Hz, notch filter 60 Hz, paper speed 30 mm/s, periodic bursts of bilateral synchronic generalized sharp wave—slow wave complexes, spike-and-wave complexes and polyspikes (fragmented aspect of hypsarrhytmia) were registered. Abbreviations: EEG—electroencephalography, LFF—low frequency filter, HFF—high frequency filter.

Subsequently, within five days, a new type of seizures appeared. Series of 3–40 paroxysmal events with eye deviation and brief jerking in the arms—IS—repeated 3–5 times per day. In EEG recorded at drowsiness, bursts of bilateral synchronic usually generalized sharp wave—slow wave complexes, spike-and-wave complexes and olyspikes with amplitude up to 370 μV alternated with periods of attenuation. Hypsarrhythmia has been reported (Figure 4).

IESS was recognized, vigabatrin treatment and vitamin B6 were introduced. IS disappeared within a few days after treatment modification. The result of the panel of the NGS epilepsy gene was obtained; however, only two variants were reported: likely pathogenic (LP) in the dihydropyrimidine dehydrogenase deficiency (DPYD) gene, (inheritance pattern: autosomal recessive, heterozygous) and pathogenic (P) in the transfer ribonucleic acid methyltransferase 5 (TRMT5) gene (inheritance pattern: autosomal recessive, heterozygous). The presence of a variant on a single allele of a gene associated with recessive disease may indicate carrier status. Typically, carriers of these variants do not exhibit clinical symptoms (Table 1A). Information on the topic of genetic diagnostics [9,10,11,12,13,14,15,16,17,18,19,20,21,22,23] can be found in Table 1B.

After a while, at the age of 7 months, the child presented a fever-associated seizure at the local hospital. Urinary tract infection was diagnosed and cefuroxime treatment was started. However, extreme irritability and short (1–2 min) multiple episodes per day of generalized tremor with crying in wakefulness soothed by hugging appeared. Some regression and stagnation of development became obvious now. The girl could not support herself on the hands in the prone position anymore, but only on the forearms. She was able to turn from the supinated to the prone position, through the left side. An exhaustive work-up was done at that point. Cell count, protein and lactate levels in the Cerebrospinal Fluid (CSF) study were normal. Polymerase Chain Reaction (PCR) studies of CSF for infection with Escherichia coli, Haemophilus influenzae, Listeria monocytogenes, Neisseria meningitidis, Streptococcus agalactiae, Streptococcus pneumoniae, Cytomegalovirus, Enterovirus, Herpes simplex virus 1, Herpes simplex virus 2, Human herpesvirus 6, Varicella-zoster virus, Human parechovirus, Cryptococcus were negative. The microbiological tests of blood and CSF were negative. The panel of autoimmune encephalitis antibodies directed against the N-Methyl-D-Aspartate Receptor (NMDAR), α-Amino-3-Hydroxy-5-Methyl-4-Isoxazolepropionic Acid Receptor Subunits 1 and 2 (AMPA 1/2), Contactin-Associated Protein-like 2 (CASPR2), Dipeptidyl Aminopeptidase-like Protein 6 (DPPX), gamma-aminobutyric Acid Type B Receptor Subunits 1 and 2 (GABAR B1/B2) in serum and CSF was negative. The onconeuronal antibody panel in blood and CFS revealed a borderline antibody titter against Recoverin (RCVRN). Antibody studies against the Myelin Oligodendrocyte Glycoprotein (MOG) protein and Aquaporin 4 in serum were negative. The enzyme test for Aromatic L-Amino Acid Decarboxylase Deficiency (AADCD) was negative. Bilateral synchronic sharp wave—slow wave complexes over temporo-parietal leads with amplitude up to 70mcV were reported in sleep EEG, but there was no evidence of hypsarrhythmia anymore. Brain MRI with contrast enhancement was repeated. Treatment with vigabatrin, valproic acid, and vitamin B6 was sustained.

**Figure 5:**
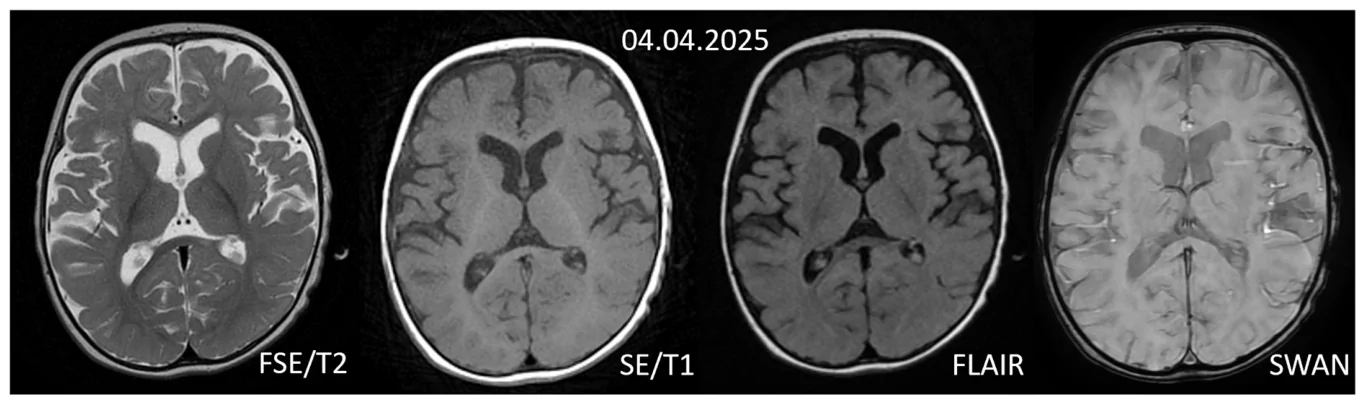
Brain MRI figures performed at the age of 8 months (4 April 2025): FSE/T1, SE/T2, FLAIR and SWAN sequence images from the examination of 4 April 2025 showing normal signal of brain structures, including both thalami and internal capsules. The examination was performed with a GEMS Artist 1.5T device. Abbreviations: MRI—Magnetic Resonance Imaging, FSE/T1—Fast Spin Echo/T1-Weighted, SE/T2—Spin Echo/T2-Weighted, FLAIR—Fluid-Attenuated Inversion Recovery, SWAN—Susceptibility-Weighted Angiography.

During the next 3 weeks, the patient has experienced 2–3 days periods of extreme irritability alternated with periods of apathy and sleepiness of similar duration. The parents claimed that the daughter lost her ability to crawl. Finally, at the age of 8 months the girl was admitted to the clinic with signs mentioned at the beginning of this report. On admission, she was extremely sleepy, her muscle tone was very low, dystonic movements and action tremor were seen in her forearm and hands. Sleep EEG was performed: normal sleep patterns were preserved, only rare bilateral synchronic and asynchronic sharp waves and sharp wave—slow wave complexes were registered over centro-temporal leads. Brain MRI revealed an increase in MR signal density in DWI with diffusion restriction in ADC bilaterally in the thalami and the lower part of both internal capsules; and dilatation of subarachnoid spaces and ventricular system (Evans ratio—0.33) (Figure 1). In the remaining sequences, changes in the brain parenchyma were not visible (Figure 5).

Inborn errors of metabolism (Leigh disease, methylmalonic aciduria), encephalitis, infection-triggered encephalopathy, and toxic vigabatrin-induced encephalopathy were considered in the differential diagnosis. The respiratory panel was positive for rhino/enterovirus and parainfluenza virus infection. The CSF study was normal. The PCR study of CSF for common neurotropic pathogens was repeated and was negative. The level of carnitine in blood serum was reduced. The levels of ammonia, methylmalonic acid, lactate in serum, and lactate in CSF were normal. A tandem mass spectrometry panel of amino acids and acylcarnitine in serum was performed. No metabolic profile indicative of congenital metabolic disorders was demonstrated. Tests for congenital disorders of glycosylation (transferrin isoforms), adenyl succinate lyase deficiency (ADSLD) and adrenoleukodystrophy (Very Long-Chain Fatty Acids—VLCFA level in serum) were negative.

The differential diagnosis considered secondary carnitine deficiency caused by valproic acid intake, which is why serum carnitine levels were measured. The tandem mass spectrometry panel of amino acids and acylcarnitines in serum also indicated the possibility of secondary carnitine deficiency. The presence of the metabolite C8-dicarboxylic acylcarnitine (C8DC) was detected, which is associated with valproic acid intake. As is known, VPA inhibits the biosynthesis of carnitine by decreasing the concentration of alpha-ketoglutarate and may contribute to carnitine deficiency [24].

The whole-exome sequencing (WES) study had been ordered (detailed information [9,10,11,12,13,14,15,16,17,18,19,20,21,22,23] in Table 1B). Vigabatrin was discontinued. Treatment with pulses of Solu-Medrol (25 mg/kg—5 days), Acyklovir (7 days), Ceftriaxon (5 days) and Oseltamivir (5 days) was introduced. Stepwise improvement of the child’s condition was observed with rapid resolution of sleepiness and reacquisition of lost motor milestones (ability to crawl and support on hands in prone position); however, cessation of movement disorder lasted a bit longer. The treatment plan was further modified with the introduction of levetiracetam and clobazam.

**Figure 6:**
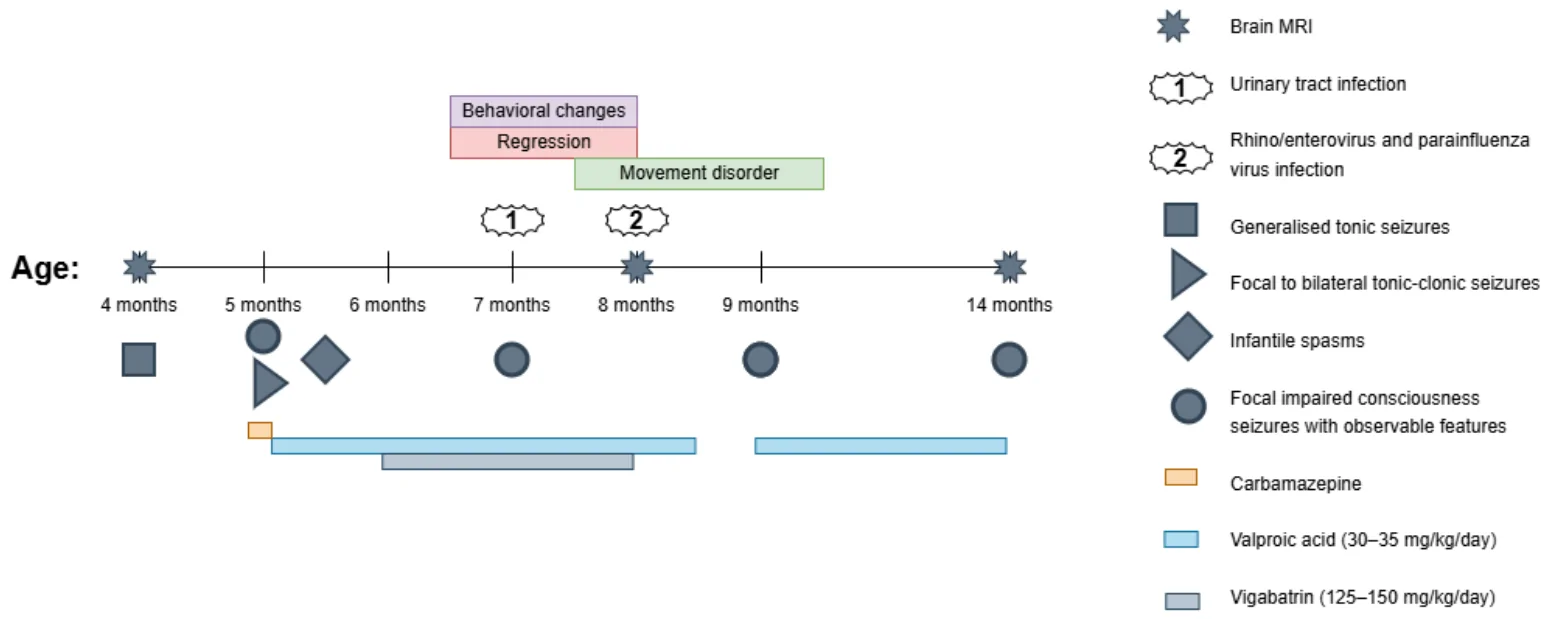
A graphical analysis of the case.

Finally, the result of the WES study was received. It revealed a new potentially pathogenic variant in one allele of the PCDH19 gene—c.1072del (p. Val358SerfsTer10). In addition, two variants of uncertain significance (VUS) were reported. The first is in the Potassium Voltage-Gated Channel Subfamily Q Member 2 (KCNQ2) gene associated with benign neonatal seizures with autosomal dominant mode of inheritance, developmental and epileptic encephalopathy 7 and myokymia, also with autosomal dominant mode of inheritance. The second is in the SIX Homeobox 5 (SIX5) gene associated with brachio-oto-renal syndrome 2 with autosomal dominant mode of inheritance (Table 1A). In our patient’s case, the PCDH19 gene mutation was considered the causative mutation, while the other mutations were considered incidental findings. A graphical analysis of the case is presented in Figure 6.

At further follow-up at the age of 14 months complete resolution of the movement disorder was reported. The girl was also able to sit and stand up on her own. The frequency and intensity of seizures have decreased. The interictal EEG at sleep was normal. The control brain MRI revealed regression of previously reported changes in both the thalami and the lower part of both internal capsules (Figure 1).

## 3. Discussion

The novel heterozygous frameshift mutation in the PCDH19 gene (c.1072del; p.Val358SerfsTer10) provides a definitive genetic etiology for the core features observed in our patient. On the one hand, the mutation in the PCDH19 gene could partially explain the clinical picture seen in the reported patient. Seizure onset before one year of age, normal development, and neurologic examination at the onset of seizures and seizure clustering are signs that correlate with the diagnosis of PCDH19-clustering epilepsy [25,26]. In addition, focal seizures with impaired consciousness similar to those observed in the reported patient with tonic extension of the upper arms, deviation of the head and eyes, pallor of the face, expression of fear, and screaming were reported in the half of the patients with PCDH19-clustering epilepsy [26,27]. On the other hand, such features as IS, subacute development of movement disorder, hypsarrhythmia in EEG, changes in signals from deep grey matter structures, and brain stem in brain MRI study are uncommon for patients with PCDH19-clustering epilepsy [5,28,29,30,31,32]. The exceptionally rare initial presentation for the PCDH19 manifestation: IS and subsequent development of IESS explain the severe down-regulation of neurosteroid-mediated GABAergic inhibition—recently highlighted in PCDH19 pathophysiology [3,30]. This condition is often drug-resistant and characterized by a highly variable response to antiseizure treatment. Levetiracetam, especially when initiated early, showed the greatest reduction in seizures, followed by clobazam and potassium bromide. The response to steroid therapy is variable. Ganaxolone has shown promise in recent studies. Vagus nerve stimulation, ketogenic diet, and temporal lobectomy have been shown to reduce the number of seizures in some cases [5]. Initial antiseizure therapy offers further retrospective validation of this pathogenic variant. It is established that sodium channel blockers, such as carbamazepine, are ineffective or even detrimental in this population [5,33]. Rapid cessation of status epilepticus and successful resolution of the cluster following intravenous valproic acid administration perfectly mirror the molecular treatment responses established [5,33].

Vigabatrin is a chemical analogue of GABA and acts primarily as an irreversible inhibitor of the GABA transaminase enzyme (GABA-T) [34,35]. If it comes to movement disorder, subacute development and abnormalities reported on brain MRI, it is notable that these signs appeared after starting vigabatrin treatment and ceased after its discontinuation. It is already known that the percentage of VABAMR among vigabatrin-treated patients ranges between 15% and 47% according to different studies [7]. The specific neuroradiological profile documented in our patient, characterized by limited water diffusion and cytotoxic edema of the thalamus and lower part of both internal capsules, strongly reflects the picture of VABAMR already reported in the literature [34,35,36,37]. Lesions may also be bilaterally located in the globus pallidus, central tegmental tracts, and dentate nuclei [38].

This phenomenon involves transient intramyelin edema caused by excessive accumulation of GABA in areas with high receptor density [7]. The subacute development of movement disorders observed in this case has also previously been associated with these specific limitations of thalamic and subcortical diffusion [37,38,39]. VABAMR appears to be mostly asymptomatic in vigabatrin-treated patients and usually disappears after discontinuation of treatment. Furthermore, this pattern is not unique to patients treated with vigabatrin [6,7,35,40]. Massive intracellular accumulation of GABA induced by vigabatrin triggers profound shifts in cellular osmolarity and energy utilization [34]. Several convergent risk factors in this case point directly to acquired drug-induced etiology (Table 2). Infantile age (under 11/12 months) and the physiological demands of active myelination make these deep structures uniquely vulnerable to vigabatrin-induced intramyelinic edema [34,35,37]. It should be noted that our patient was an infant during vigabatrin treatment. Our patient was treated with vigabatrin at a dose of 125–150 mg/kg/day. This means that the dose used was below the risk of toxicity threshold [41]. Concomitant administration of systemic steroids in conjunction with high-dose vigabatrin represents a critical, clinically documented trigger to transition asymptomatic neuroimaging anomalies to acute, symptomatic neurotoxicity [35,37,42]. Due to the child’s serious condition, in addition to discontinuing vigabatrin, broad-spectrum therapy was implemented, including antimicrobial and immunomodulatory therapy (methylprednisolone). The child’s condition improved, and the movement disorders resolved. It is impossible to predict whether the changes would have resolved sooner or later without methylprednisolone—this approach cannot be recommended.

It can be suggested that the biochemical depletion of carnitine in our patient theoretically acted as a pivotal metabolic multiplier for this iatrogenic vulnerability. Cytotoxic edema results from a rapid decrease in Adenosine Triphosphate (ATP) in the cell, leading to failure of the ion pump [43]. Carnitine deficiency reduces mitochondrial energy-buffering capacity [44]. Carnitine enables the transport of long-chain fatty acids into mitochondria, where they are burned through beta-oxidation. This process produces sustained ATP accumulation [45]. When this process is impaired, the phosphocreatine system cannot keep up with ATP regeneration [46]. Susceptibility to cytotoxic cerebral edema increases [47]. Also, vigabatrin is a potential trigger for cerebral edema [48].

Our patient had a polyetiological viral infection during exposure to vigabatrin. The occurrence of infection during vigabatrin exposure did not appear to predispose patients to VABAMR [49]. There is no evidence in the available literature that heterozygous mutations in the PCDH19 [3], DPYD [50], TRMT5 [51], SIX5 [52], or KCNQ2 [53,54] genes increase vigabatrin neurotoxicity. DPYD variants raise toxicity risks only upon exposure to fluoropyrimidine-based chemotherapeutics [50]. In clinical practice, voltage-dependent sodium channel blockers are used with good results in antiseizure treatment in patients with KCNQ2 mutations [53,54]. In our case, carbamazepine proved ineffective as an anti-seizure treatment.

Based on the differential diagnosis [6,38], entities of the disease with similar changes in MRI were excluded, which confirms the toxicity of vigabatrin. The literature contains detailed studies on the process of differentiating diseases with bilateral MRI changes in the basal ganglia. These lesions are hyperintense in T2-weighted sequences, as well as those associated with diffusion water restriction. Similar patterns of MRI changes in VABAMR include Krabbe disease, deficiency of the pyruvate dehydrogenase complex (PDHC), mitochondrial translation disorder, and Kearns–Sayre syndrome. Based on the MRI of the brain, four different patterns (clusters) were described and disease entities were assigned to them. VABAMR and Krabbe disease were assigned to a common pattern described as Cluster 3 [38]. In our patient’s case, hyperintense lesions in T2-weighted sequences were not observed on MRI. Based solely on the MRI, we were unable to determine that it was WABAMR. Metabolic and genetic studies did not confirm these diagnoses. The WES did not detect a homozygous, pathogenic mutation in the GALC gene [55].

The authors recognize that functional and metabolic neuroimaging methods in children play a key role in localizing epileptogenic foci, mapping neural network disorders, and qualifying for neurosurgical procedures, e.g., functional Magnetic Resonance Imaging (fMRI), positron-emission tomography (PET), low-resolution electromagnetic tomography (LORETA), single photon emission spectroscopy (SPECT), near-infrared spectroscopy (NIRS), and optical imaging of intrinsic signals (IOS) [56]. However, similar studies were not performed in our patient. In the literature, there are examples of use of the quantitative MRI to investigate the cortex and white matter in 20 PCDH19-mutated patients. It was found that patients exhibited bilateral reductions in local gyrification index (lGI) in limbic cortical areas, including the parahippocampal and entorhinal cortex and the fusiform and lingual gyri, and altered diffusivity features in the underlying white matter. In patients with an earlier onset of seizures, worse psychiatric manifestations and cognitive impairment, reductions in lGI, and diffusivity abnormalities in the limbic areas were more pronounced [57].

Until now, there have only been few reports of movement disorders [58,59]. In November 2009, the UK Medicines and Healthcare Products Regulatory Agency suggested updating the summary of product characteristic and the patient information leaflet with a warning about this issue. Therefore, in the event that a new movement disorder occurs during vigabatrin treatment, a dose reduction or a gradual discontinuation of treatment should be considered [60]. This strategy was successfully adopted in the reported patient. Recognizing this fully reversible etiology [61] prevents these symptoms from being incorrectly attributed to PCDH19 cluster epilepsy, childhood bilateral basal ganglia disorders, including systemic metabolic crises [3].

## 4. Conclusions

It is common practice in child neurology that treatment choices are made in circumstances where the real cause of the disease is unknown or is still unknown. The consideration of even rare side effects of drugs is important. Sometimes, as was seen in the case presented, the side effects of the drugs could imitate the natural course of severe neurological diseases. The doctor’s awareness of these side effects could save them from treatment mistakes and preserve patient health. Furthermore, appropriate early and expanded genetic testing could potentially simplify and shorten the diagnostic journey in many cases, especially in the early childhood patient group with epilepsy.

## Figures and Tables

**Table 1:** (**A**). List of single nucleotide variants detected. (**B**). Detailed information on the metabolic assumptions of genetic testing [9,10,11,12,13,14,15,16,17,18,19,20,21,22,23].

**(A). List of single nucleotide variants.**									
**No. Variant**	**Gene**	**HGVS Nomenclature (Transcript, Protein Change; Chromosome, Position)**	**Phenotype MIM nr (OMIM) and Inheritance**	**Population Frequency**	**dbSNP**	**ACMG**	**ClinVar (as for 16.07.25)**	**Type of Variant**	**Genotype**
1	*PCDH19*	NM_001184880.2:c.1072del NP_001171809.1:p.Val358SerfsTer10) ChrX(hg38):100407525AC>A	Developmental and epileptic encephalopathy 9 (300088), XL	Novel	novel	LP	novel	frameshift	het.
2	*DPYD*	NM_000110.4:c.187A>G NP_000101.2:p.Lys63Glu Chr1(hg38):97828160T>C	5-fluorouracil toxicity (274270), AR dihydropyrimidine dehydrogenase deficiency (274270), AR	0.0032%	rs367619008	LP	yes	missense	het.
3	*TRMT5*	NM_020810.3:c.312_315del NP_065861.3:p.Ile105fs Chr14(hg38):60979582CTATT>C	Peripheral neuropathy with variable spasticity, exercise intolerance, and developmental delay (616539), AR	0.1128%	rs755184077	P	yes	frameshift	het.
4	*KCNQ2*	NM_172107.4:c.2608C>T NP_742105.1:p.Pro870Ser Chr20(hg38):63406655G>A	Developmental and epileptic encephalopathy 7 (613720), AD Myokymia (121200), AD seizures, benign neonatal, 1 (121200), AD	NA	rs2516089887	VUS	yes	missense	het.
5	*SIX5*	NM_175875.5:c.1073C>G NP_787071.3:p.Pro358Arg Chr19(hg38):45766886G>C	Branchiootorenal syndrome 2 (610896), NA	NA	rs1344313680	VUS	no	missense	het.
No.—Number, HGVS—Human Genome Variation Society, MIM nr—Phenotype Mendelian Inheritance in Man number, OMIM—Online Mendelian Inheritance in Man, dbSNP—Database of Single Nucleotide Polymorphisms, ACMG—American College of Medical Genetics and Genomics, *PCDH19*— Protocadherin 19, *DPYD*—Dihydropyrimidine Dehydrogenase Deficiency, *TRMT5*—Transfer Ribonucleic Acid Methyltransferase 5, *KCNQ2*—Potassium Voltage-Gated Channel Subfamily Q Member 2, *SIX5*—SIX Homeobox 5, P—pathogenic, LP—likely pathogenic, VUS—variant of uncertain or unknown significance; AR—autosomal recessive; AD—autosomal dominant; XL—X-linked; NA—not available, het.—heterozygous									
**(B). Metabolic assumptions of genetic testing** [9,10,11,12,13,14,15,16,17,18,19,20,21,22,23].									
➢ A peripheral blood sample was taken from the patient. ➢ Genomic Deoxyribonucleic Acid was extracted using the Maelstrom 4800 automated nucleic acid extraction system. NGS and WES were performed in the proband using the Twist Human Core Exome Plus Kit (Twist Bioscience) and sequenced with Illumina technology (100× depth of mean coverage). The reads obtained were aligned to the human genome 38—reference genome (developed by the Genome Reference Consortium 38, GRCh38). The Quality Control (QC) value obtained was <99% for Quality Score 30 (Q30). Alignment and variant calling were performed with an in-house bioinformatics pipeline. Raw sequencing reads were assigned to the human reference genome GRCh38 assembly using BWA MEM (bwa mem2.avx2 mem 0.7.17-r1188) [9]. ➢ Duplicates were removed using biobambam2 version 2.0.183 [10]. ➢ Variants were called using HaplotypeCaller (GATK v4.2.6.1) [11,12] and FreeBayes v1.3.2 [13], and named using Variant Effect Predictor (VEP115) [14]. ➢ To estimate pathogenicity, data were extracted from multiple genetic databases, including Clinical Variants (ClinVar), Database of Single Nucleotide Polymorphisms (dbSNP), DatabasE of genomiC varIation and Phenotype in Humans using Ensembl Resources (Decipher), Clinical Genome Resource (ClinGen), Online Mendelian Inheritance in Man (OMIM) [15,16,17,18,19]. ➢ The prevalence of variants in control populations was checked in the 1000 Genomes [20] and Genome Aggregation Database—Version 4 (gnomAD—v4, Broad Institute) databases [21]. ➢ In silico splicing analysis was also performed using algorithms embedded in Alamut Visual Plus v2.1 software (Sophia Genetics). The variants were classified. Pathogenicity was evaluated in accordance with international recommendations and criteria. In this study, the American College of Medical Genetics and Genomics (ACMG) Standards and Guidelines for the interpretation of sequence variants were used [22]. ➢ A 1% frequency filtering criterion was applied. The eXome Hidden Markov Model (XHMMv1.0) algorithm and in-house scripts were used to search for small, rare Copy Number Variations (CNVs). Although NGS studies allowed to detect variants in these studies, all of them need to be confirmed by PCR and the Sanger sequencing method for final confirmation [23]. ➢ Variant analysis focused on genes associated with the observed clinical phenotype, including seizure and neurodevelopmental epilepsy. From among identified variants, five were reported, including one novel heterozygous mutation c.1072del (p.Val358SerfsTer10) in the PCDH19 gene (NM_001184880.2) correlated with the patient’s phenotype. Considering the patient’s clinical data, the novel variant was classified as likely pathogenic, according to the ACMG variant interpretation guidelines: Pathogenic Very Strong 1 (PVS1, null variant in a gene where loss of function is a known mechanism of disease), Pathogenic Moderate 2 (PM2, extremely low frequency in gnomAD population databases/absent from controls). No CNVs were identified in the analysis of WES results.									

**Table 2:** The risk factor for Vigabatrin-Associated Brain Abnormalities on Magnetic Resonance Imaging (VABAMR).

The Risk Factor of VABAMR	Vote	Literature Source
The higher the dose of vigabatrin per kilogram of body weight per day (peak dose), the higher the risk.	Yes	[6,34,36]
The risk of VABAMR can be reduced by avoiding extremely high doses (peak dose > 165 mg/kg/day).	Yes	[33]
The risk of asymptomatic VABAMR can be reduced by avoiding extremely high doses (peak dose > 175 mg/kg/day).	Yes	[34]
No dependence of risk on the cumulative dose of vigabatrin	Yes	[34]
The use of concomitant hormonal therapy (corticosteroids and ACTH) increases the risk.	Yes	[34,40]
The risk of symptomatic VABAMR can be reduced by postponing concomitant hormonal therapy.	Yes	[33,34]
The risk is higher in infancy (<11 months old) compared to older age groups.	Yes	[33]
The risk is higher in infancy (<12 months old) compared to older age groups.	Yes	[6,36]
The risk is higher in infancy (<24 months old) compared to older age groups.	Yes	[43]
The VABAMR usually resolves after discontinuing vigabatrin after a period of 3 months	Probable	[33]
Other genetic, metabolic, and environmental (treatment) modifiers of VABAMR risk	Suspected	[34]
Duration of vigabatrin exposure	No	[43]
Infantile Spasm etiology	No	[43]
Microcephaly	No	[43]
Developmental delays	No	[43]
Being underweight	No	[43]
Concurrent use of more than three antiseizure medications	No	[43]
Concurrent use of oral steroids	No	[43]
Delayed brain myelination	No	[43]
Occurrence of infection during vigabatrin exposure	No	[43]

VABAMR—Vigabatrin-Associated Brain Abnormalities in Magnetic Resonance Imaging.

## Data Availability

The original contributions presented in the study are included in the article, further inquiries can be directed to the corresponding author.
